# Repeated Administration of Norbinaltorphimine Produces Cumulative Kappa Opioid Receptor Inactivation

**DOI:** 10.3389/fphar.2019.00088

**Published:** 2019-02-06

**Authors:** Charles Chavkin, Joshua H. Cohen, Benjamin B. Land

**Affiliations:** Department of Pharmacology, University of Washington, Seattle, WA, United States

**Keywords:** kappa opioid receptor, depression, addiction, receptor inactivation, drug development, sex differences

## Abstract

Kappa receptor activation by dynorphins contributes to the anxiogenic, dysphoric, and cognitive disrupting effects of repeated stress, suggesting that kappa receptor antagonists might have therapeutic utility in the treatment of stress disorders. Three classes of kappa antagonists have been distinguished: non-selective, selective-competitive (readily reversible), and non-competitive (receptor-inactivating); however, which would be the most effective medication has not been established. To assess the utility of receptor inactivating antagonists, we tested the effects of a range of doses in both male and female mice. As previously established, the antinociceptive effects of the kappa agonist U50,488 were blocked by a single injection of the long-acting antagonist norbinatorphimine (norBNI) (10 mg/kg i.p.) in male mice. Ten to 20-fold lower doses of norBNI were ineffective after a single administration, but daily administration of 1.0 or 0.5 mg/kg for 5 days completely blocked U50,488 antinociceptive effects. Daily administration of 0.1 mg/kg norBNI produced slowly accumulating inhibition and completely blocked the antinociceptive effect of U50,488 after 20–30 days. Estrogen reduces female sensitivity to kappa opioid effects, but 30 days of 0.1 mg/kg norBNI completely blocked U50,488 analgesia in ovariectomized mice. Receptor inactivation in both male and female mice treated for 30 days with 0.1 mg/kg norBNI persisted for at least 1-week. These results suggest that receptor-inactivating kappa antagonists are effective in both males and females when given at 100-fold lower doses than typically administered in preclinical studies. The enhanced safety of this low-dosing protocol has important clinical implications if receptor inactivating kappa antagonists advance in medication development.

## Introduction

The physiological stress response provides important metabolic and cardiovascular adaptations that protect the organism and enhance survival; however, sustained stress exposure can produce pathophysiological effects that are ultimately deleterious (Bale and Vale, 2004). For vulnerable individuals, chronic stress can produce mood disorders and increase the risk of substance use disorder (Sinha et al., 2011), and medications able to promote stress-resilience might have important therapeutic utility in the treatment of depression, anxiety, and drug addiction. One type of stress-resilience medication that is currently under clinical development are the kappa opioid receptor antagonists, which block the effects of the endogenous dynorphin opioid peptides (Carroll and Carlezon, 2013). Dynorphins are released by corticotropin releasing factor (CRF) in brain during stress exposure and encode the aversive, anxiogenic, and dysphoric responses to stress (Bruchas et al., 2010). Clinical trials with kappa antagonists show promise (Chavkin and Koob, 2016), but important questions are necessary to resolve at the preclinical level to guide medication development. Specifically, it is not clear which type of kappa antagonist would be most effective. Three classes have been distinguished: (1) ***non-selective antagonists*** that bind to other receptors besides kappa (e.g., buprenorphine, naltrexone, naloxone, and nalmefene), (2) short-acting ***selective*** ***competitive antagonists*** that specifically inhibit kappa receptor activation [e.g., PF-04455242, LY2456302 (also called CERC-501, JNJ-67953964), and BTRX-335140], or (3) kappa selective***receptor-inactivating*** antagonists that produce a long-lasting structural change in the kappa receptor signaling complex by a recently defined c-Jun Kinase mechanism (e.g., norBNI, JDTic, and GNTI) (Schattauer et al., 2017). The latter can be considered as non-competitive antagonists or as a novel class of functionally selective agonists.

There are theoretical advantages and disadvantages of each class of kappa antagonists as potential therapeutics. Receptor-inactivating kappa antagonists may produce more stable stress-resilience, but JNK activation may also produce adverse effects and long-duration of effect would make dose titration problematic. In the present study, we asked if the receptor-inactivating class of kappa antagonist can produce long-acting receptor inactivation in a dose-dependent manner for both male and female mice. Our conjecture is that receptor inactivating kappa antagonists might be safer and more effective than non-selective or competitive kappa antagonists if they can be administered at very low doses that produce accumulating receptor inactivation with minimal off-target effects. In this proof-of-principle study, we found that daily norbinaltorphimine (norBNI) administration at 100-fold lower doses than required for acute receptor antagonism completely blocked kappa receptors in both male and female mice.

## Methods

### Drugs

Norbinaltorphimine (norBNI) and U50,488 (NIDA Drug Supply program) were dissolved in sterile saline (0.9%) at injected at 10 mL/kg (i.p.). The GRK2/3 inhibitor CMPD101 (Tocris Bioscience) was used as described (Abraham et al., 2018).

### Subjects

Male and female C57BL/6N mice (*n* = 66) ranging from 8 to 16 weeks of age were used. All experimental procedures were approved by the University of Washington Institutional Animal Use and Care Committee. All testing was during the light phase of the 14-h light/dark cycle. Ovaries were removed from female mice under isoflurane anesthesia as previously described (Smith et al., 2005). Mice were allowed 2 weeks recovery prior to behavioral experiments.

### Tail-Flick Analgesia

Mice were administered norBNI or an equivalent volume of saline daily at the specified dose (0.1–10 mg/kg, i.p.) and for the specified duration (1–30 days). 24 h after the norBNI injection on day 2, 6, 11, 21, and 31, the response latency for the mouse to withdraw its tail following immersion into 52.5 +/- 0.2°C water was measured before and 30 min after U50,488 administration (10 mg/kg, i.p.). One additional U50,488 test was performed on day 37, 1 week after the last norBNI injection. Data are expressed as maximum possible effect, normalized to time matched saline/U50,488 controls.

### Statistics

Group differences were determined using one-way ANOVA and *post hoc* comparisons analyzed with Dunnett’s multiple comparison test (comparison group = saline), or linear trend with α set to 0.05. Data were analyzed with GraphPad Prism 7.

## Results

**FIGURE 1:**
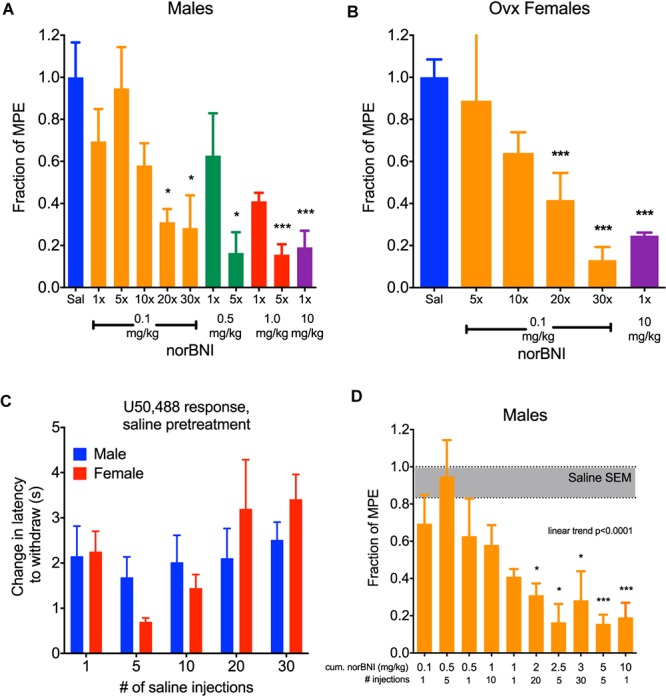
Daily administration of low doses of norBNI produced accumulating receptor inactivation. **(A)** Male C57BL/6 mice were injected with norBNI at the doses and number of daily administrations listed on the *x*-axis and the fraction of the maximal possible analgesic effect in the tail flick latency assay (MPE, determined from saline-pretreated animals) after U50,488 (10 mg/kg) is shown on the *y*-axis; (*n* = 4–18, ANOVA *F* = 4.377, ^∗^*p* < 0.05, ^∗∗∗^*p* < 0.001) **(B)** Ovariectomized female C57BL/6 mice were injected with norBNI at the doses and number of daily administrations listed on the *x*-axis and the fraction of the MPE of tail flick latency was determined after U50,488 (*n* = 4–5, ANOVA *F* = 17.16, ^∗∗∗^*p* < 0.001) **(C)**. The analgesic effect of U50,488 does not significantly change over the course of the experiment in either males or females. *Y*-axis represents the change in latency to remove tail post-U50,488 (10 mg/kg) subtracted from the latency pre-U50,488; (*n* = 3–8, 2-way ANOVA *F*_interaction_ = 0.9131, *p* = 0.47; *F*_day_ = 2.21, *p* = 0.09; *F*_,sex_ = 0.06, *p* = 0.80) **(D)**. Cumulative dosing of norBNI in males shows that significant effects are apparent at 2 mg/kg equivalence. Bars are reorganized from panel A in increasing cumulative dosing of norBNI; (*n* = 4–18, ANOVA *F* = 4.377, *post hoc* for linear trend).

In a typical preclinical study using norBNI, a single injection of 10 mg/kg (i.p.) is administered to the experimental animal to completely inhibit kappa opioid responses. We confirm that when male C57BL/6 mice were given this dose and challenged 24 h later with 10 mg/kg U50,488 (a selective KOR agonist), the analgesic response to U50,488 was fully blocked (Figure 1A). For mice given a 10- or 20-fold lower dose of norBNI (1 or 0.5 mg/kg), the response 24 h later to U50,488 was only partially blocked in a dose-dependent manner. However, if male mice are given 1 or 0.5 mg/kg norBNI daily for 5 days, the U50,488 response was fully blocked. When the dose of norBNI was lowered still further and the mice were injected daily with 0.1 mg/kg norBNI, the analgesic response to U50,488 challenge was slowly reduced and was fully blocked only after 20–30 days of dosing.

Female mice and rats have been reported to be less sensitive than males in their responses to kappa opioid agonists and antagonists (Russell et al., 2014; Abraham et al., 2018; Laman-Maharg et al., 2018; Williams and Trainor, 2018). These sex differences raise important questions about the utility of kappa antagonists in the treatment of stress-disorders in women. Additionally, U50,488 treatment does not produce analgesia in female mice when their estrogen levels are high. Estrogen was found to activate G protein Receptor Kinase 2 (GRK2), which blocked Gβγ mediated analgesia (Abraham et al., 2018). Similar to results using kappa agonists, female mice respond to receptor inactivating KOR antagonists in an estrogen-sensitive manner; norBNI is not long lasting in females when administered during high estrogen phases of their estrus cycle, and inhibition of GRK2 by CMPD101 (15 mg/kg, 30 min prior) made 10 mg/kg norBNI long-lasting (data not shown). After ovariectomy, female mice respond to the analgesic effects of U50,488 similarly to males. In this study, U50,488 (10 mg/kg) produced significant antinociceptive effects in female mice that were blocked by a single injection of 10 mg/kg norBNI (Figure 1B). Consistent with the effects observed with males, daily administration of 0.1 mg/kg norBNI also significantly blocked U50,488 effects after 20–30 days. In this protocol, mice were each injected with U50,488 five times: first 24 h after the single norBNI injection (1×), then after 5, 10, 20, and 30 days. Daily treatment of ovariectomized females with 0.1 mg/kg norBNI fully blocked U50,488-induced analgesia along the same time course as males. There was no evidence of analgesic tolerance to U50,488 caused by this injection protocol; the response to U50,488 did not significantly change during the course of the injection series for either male or female mice (Figure 1C).

As expected in this dosing protocol, the effects of norBNI accumulated with each injection. This result is predicted by the long effective duration of norBNI effects, which have been shown to reverse with a half-life of approximately 14 days in male mice (Bruchas et al., 2007). To visualize this cumulative effect, we replotted the blockade of analgesia as the total amount of norBNI injected at the time of U50,488 administration (Figure 1D). The degree of kappa receptor inhibition clearly depended on the history of norBNI administration, following a linear trend, with a cumulative dose of about 2 mg/kg as the point where significant block of analgesia is produced.

**FIGURE 2:**
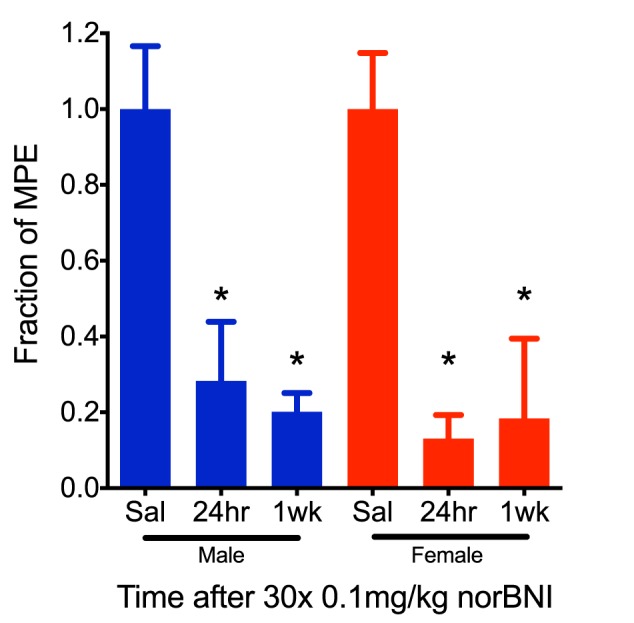
The effects of repeated, low-dose norBNI are long-lasting. Male and female mice were tested 24 h and 1 week after the last of 30 daily injections of 0.01 mg/kg norBNI or saline. *Y*-axis represents the fraction of the MPE in the tail flick latency assay after U50,488 (10 mg/kg) [*n* = 4–9, ANOVA *F* = 5.234 (male), *F* = 5.437 (female), ^∗^*p* < 0.05].

To further characterize the effects of this dosing scheme, we challenged mice with U50,488 1 week after receiving daily low doses of norBNI (0.1 mg/kg) for 30 days and found that in both male and female mice the kappa receptors remained inactivated (Figure 2). Thus, clinical efficacy may be achieved at doses 100-fold lower than required for acute receptor saturation, and both males and ovariectomized female mice exhibited long-duration of kappa receptor inactivation after administration of low doses of norBNI.

## Discussion

The principal findings of the study are that receptor-inactivating kappa antagonists may be effective at 100-fold lower doses than required for acute antagonism. While sex differences are important to consider in kappa receptor directed medications, receptor inactivation by norBNI was evident in both male and female mice. The key implication of this study is that receptor inactivating antagonists may be more selective and safer medications when used at these low doses. The 3–4 weeks required for optimization of effect would not be expected to be a significant limitation for clinical treatment since antidepressants and other mood stabilizing medications often require slow titration and months of treatment before clinical efficacy is established. Whether c-Jun Kinase activation by receptor-inactivating antagonists is a safety concern is not yet clear, but it should be noted that kappa opioid receptor activation by dynorphin and mu opioid receptor activation by morphine also activate c-Jun Kinase (Schattauer et al., 2017) without documented adverse effects. Thus, physiological levels of c-Jun Kinase activation at the low pharmacological doses necessary may not have the toxic effects noted with intense activation (Bubici and Papa, 2014).

There are theoretical advantages and disadvantages of each class of kappa antagonists as potential therapeutics: non-selective antagonists have the advantage that their safety has been established by a long clinical history, and they are already FDA approved for human use. Non-selective medications can be more effective in treatment of psychiatric disorders (e.g., clinically effective antipsychotic medications lack receptor selectivity), and this may also be true in stress-disorders if resilience requires the block of multiple stress systems. However, the off-target effects of non-selective antagonists might be problematic or unnecessary. Kappa selective, competitive antagonists are conventional medications whose selectivity would theoretically reduce their off-target effects. The short acting effects could be readily titrated by dose adjustment. However, a significant concern with competitive antagonists is that they may lack clinical efficacy if they are not given at doses that adequately block the kappa receptors during an unanticipated stress event. High antagonist doses (resulting in off-target effects) and frequent dosing (resulting in challenging medications compliance issues) may be required to block stress-induced dynorphin effects. In contrast, receptor-inactivating kappa antagonists may produce more stable stress-resilience with off-target effects minimized by the low doses required. While the utility of receptor inactivating kappa antagonists in women remains to be established, we predict that these drugs would be effective during the normal menstrual cycle while estrogen levels are low and that kappa receptor inactivation will accumulate with daily administration of low doses of antagonist. In support of this conjecture, the recovery rate of kappa receptors in rhesus monkeys treated with norBNI required more than 20 weeks (Ko et al., 2003), suggesting that receptor half-life in primates is even longer than rodents.

Therapeutic efficacy of antagonists requires adequate receptor blockade to prevent the endogenous agonist effects. While the degree of receptor block required for kappa system is not yet known, clinical trials of conventional competitive CRF R1 receptor antagonists failed to demonstrate efficacy (Spierling and Zorrilla, 2017), and we suggest that this may have been a consequence of R1 receptor occupancy by the antagonist doses used that was insufficient to block endogenous CRF effects. Because dynorphin is a highly efficacious agonist and spare kappa receptors are evident (Chavkin and Goldstein, 1981), dynorphin may be able to produce robust dysphoric effects at very low receptor occupancy. If true, complete kappa receptor inactivation may be necessary for clinical efficacy. This remains to be established in human stress disorders, but is an important aspect to consider in medication development. Unlike the CRF – CRF R1 receptor system where complete inactivation would produce Addison’s Disease-like symptoms, complete kappa-receptor inactivation does not affect viability (as demonstrated by the viability of prodynorphin or kappa receptor gene deletion).

The present study was done in mice and using tail flick antinociception assay as a readout. This is useful in a proof of principle study; however, receptor-inactivating antagonists are also known to have long durations of effect in the preclinical models for anxiety, depression, and addiction (Carroll and Carlezon, 2013). Thus, the results of this study support the conjecture that low doses of kappa-receptor inactivating antagonists may show greater safety, selectivity, and clinical efficacy than the alternatives.

## Ethics Statement

All experimental procedures were approved by the University of Washington Institutional Animal Use and Care Committee and were conducted in accordance with National Institutes of Health (NIH) “Principles of Laboratory Animal Care” (NIH Publication No. 86-23, revised 1985).

## Author Contributions

CC designed the experiment and wrote the paper. JC performed the experiment. BL analyzed the data and generated the figures.

## Conflict of Interest Statement

The authors declare that the research was conducted in the absence of any commercial or financial relationships that could be construed as a potential conflict of interest.
